# Nature vs. Nurture: Evidence for Social Learning of Conflict Behaviour in Grizzly Bears

**DOI:** 10.1371/journal.pone.0165425

**Published:** 2016-11-16

**Authors:** Andrea T. Morehouse, Tabitha A. Graves, Nate Mikle, Mark S. Boyce

**Affiliations:** 1 Department of Biological Sciences, University of Alberta, Edmonton, Alberta, Canada; 2 US Geological Survey, Northern Rocky Mountain Science Center, West Glacier, Montana, United States of America; Universita degli Studi di Pisa, ITALY

## Abstract

The propensity for a grizzly bear to develop conflict behaviours might be a result of social learning between mothers and cubs, genetic inheritance, or both learning and inheritance. Using non-invasive genetic sampling, we collected grizzly bear hair samples during 2011–2014 across southwestern Alberta, Canada. We targeted private agricultural lands for hair samples at grizzly bear incident sites, defining an incident as an occurrence in which the grizzly bear caused property damage, obtained anthropogenic food, or killed or attempted to kill livestock or pets. We genotyped 213 unique grizzly bears (118 M, 95 F) at 24 microsatellite loci, plus the amelogenin marker for sex. We used the program COLONY to assign parentage. We evaluated 76 mother-offspring relationships and 119 father-offspring relationships. We compared the frequency of problem and non-problem offspring from problem and non-problem parents, excluding dependent offspring from our analysis. Our results support the social learning hypothesis, but not the genetic inheritance hypothesis. Offspring of problem mothers are more likely to be involved in conflict behaviours, while offspring from non-problem mothers are not likely to be involved in incidents or human-bear conflicts themselves (Barnard’s test, *p* = 0.05, 62.5% of offspring from problem mothers were problem bears). There was no evidence that offspring are more likely to be involved in conflict behaviour if their fathers had been problem bears (Barnard’s test, *p* = 0.92, 29.6% of offspring from problem fathers were problem bears). For the mother-offspring relationships evaluated, 30.3% of offspring were identified as problem bears independent of their mother’s conflict status. Similarly, 28.6% of offspring were identified as problem bears independent of their father’s conflict status. Proactive mitigation to prevent female bears from becoming problem individuals likely will help prevent the perpetuation of conflicts through social learning.

## Introduction

“*The ideal criminal has marked peculiarities of character*: *his conscience is almost deficient*, *his instincts are vicious*, *his power of self-control is very weak…It is*, *however*, *easy to show that the criminal nature tends to be inherited”* [1]. Francis Galton, a pioneer of behavioural genetics, believed that criminal tendencies, among many other behavioural traits, were inherited, and his work sparked the long-standing nature versus nurture debate [2]. Research has now shown, however, that behaviour results from a complex interaction between an individual’s genetics and the environment [3, 4]. The question of how behaviours are developed and acquired remains an important question in behavioural ecology and is particularly important for species that often experience conflict with humans, such as large carnivores. Understanding behaviour can be challenging to address for wildlife because behavioural observations across an individual's life often are not possible; such observations, however, can provide important implications for conservation and management.

Specifically, behaviour involves decision making, which results in costs and benefits to individuals. Selecting favorable habitats, acquiring suitable food, and finding mates are all critical to an individual animal’s survival and reproduction. The acquisition of such behaviours can occur through inheritance, asocial learning, social learning, or some combination of inheritance and learning [5, 6, 7, 8]. For example, genetically based differences in foraging have been documented for a wide variety of species [8, 9, 10, 11, 12]. Alternatively, animals may develop behaviour independently through asocial learning (trial and error) [6,13]. In contrast to asocial learning, social learning occurs as a result of interacting with or observing others, usually allowing animals to acquire adaptive behaviours faster than asocial learning [6, 13, 14].

Studies of both captive and free-ranging animals have found learning to be correlated with opportunism, curiosity, behavioural plasticity, large brain size, and developed memory [15, 16, 17, 18]. Bears (Ursidae) possess each of these traits along with high maternal investment in offspring, making them predisposed to social learning [19, 20]. Although adult grizzly bears (*Ursus arctos*) are relatively asocial, grizzly bear cubs typically stay with their mother for 2–3 years [21] giving cubs opportunity for social learning from their mothers. While there is some evidence that cub behaviours are influenced by their mother’s behaviour and the habitats in which she reared her young [7, 17, 22], the data to inform the social learning question in this low-density, wide ranging species often are difficult to obtain, indirect, and of low sample size. Indeed, the literature is inconclusive on this subject and other studies did not support evidence for social learning [23]. Bears are opportunistic and flexible foragers [20, 24], and for a non-specialized species we might not expect strong evidence for social learning because a variety of options are available to meet nutritional demands–particularly in human-settled areas where anthropogenic resources are readily and easily obtained (e.g. [23, 25, 26]). If, for example, a behaviour is easily developed, social learning might not be essential to the acquisition of that behaviour [23].

Bear use of anthropogenic resources is well documented across North America. Furthermore, as grizzly bear populations expand their distribution from the high mountains after removal from much of their historic range [27, 28, 29], they increasingly overlap with human-settled lands where they are more likely to come into conflict (e.g. killing or injury of people, livestock or pets; property damage; crop damage) with human land uses such as agriculture and ranching (e.g. [30, 31, 32]). Despite the potential for conflict, recent work indicates that with supportive public opinion and effective mitigation measures, co-existence between people and large carnivores such as grizzly bears is possible [33]. Understanding how bears acquire conflict behaviours can have important management implications, especially where conflicts limit public support.

Such is the potential in southwestern Alberta where grizzly bears have been listed as a provincially threatened species under the provincial Wildlife Act since 2010 [34], but have recently been expanding [29]. Conflicts between grizzly bears and agricultural activities in the region are prevalent, increasing, and typically involve either agricultural attractants or livestock predation [32]; these incidents are slightly (55%) female biased [32]. Within Alberta, grizzly bear management frequently is reactive; Fish and Wildlife Officers often relocate problem grizzly bears in response to public complaints of conflicts [35, 36]. Adult females have the highest elasticity in grizzly bear populations [37, 38]; because the death of a breeding-age female has a more significant impact on population size than the death of a cub or sub-adult male, the provincial government has focused on reducing female mortality and keeping females with cubs within their local bear management area (BMA) to promote population growth–even if the bear has been identified as a problem individual [35, 36, 39]. Thus, the current response guidelines mandate that at a first incidence of conflict, a female with cubs will be relocated within the same BMA as opposed to males that are likely to be translocated long distances [36].

If conflict behaviour in grizzly bears is a result of social learning, then current grizzly bear management in Alberta might perpetuate the problem. By keeping problem females with cubs on the same landscape where they have been involved in conflict, cubs might be exposed to additional opportunities to learn conflict behaviours from their mother. Further, Alberta’s bear management policy might be altering demographic structure by enforcing a different mortality risk for males versus females on agricultural landscapes because translocated bears typically suffer higher mortality rates than non-translocated bears [40, 41, 42,]. Thus, acquiring conflict behaviours might be particularly maladaptive for male grizzly bears.

Here, we evaluate evidence for social learning and genetic inheritance of conflict behaviour in grizzly bears in southwestern Alberta. Using a parentage analysis, we test the prediction that conflict behaviour is genetically inherited by examining whether there is a significant relationship between the father’s conflict behaviour and offspring conflict behaviour. Conversely, if a cub learned conflict behaviour from its mother, we would predict significantly more offspring (males and females) to be involved in conflict behaviours if their mother was a problem bear.

## Study Area

**Fig 1:**
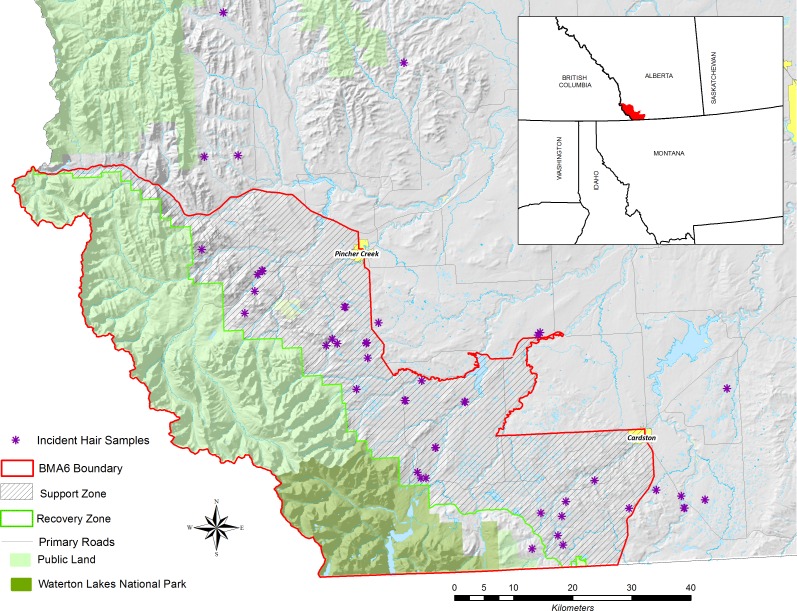
Study Area. Map of the study area (BMA 6) and incident hair samples in southwestern Alberta. An incident is defined to be an occurrence in which the grizzly bear caused property damage, obtained anthropogenic food, or killed or attempted to kill livestock or pets.

Our study area in southwestern Alberta, Canada is an area known provincially as Bear Management Area 6 (BMA 6) (Fig 1). BMA 6 was bounded by Highway 3 to the north, British Columbia to the west, Montana to the south, and the approximate edge of grizzly bear range to the east. BMA 6 includes two zones, the Recovery Zone and the Support Zone [43]. The Recovery Zone is the area in which the province explicitly intends to recover the grizzly bear population, and is predominately public land. The Support Zone in southwestern Alberta is almost exclusively private land, and is intended to support grizzly bears with home ranges that do not fall entirely within the Recovery Zone. While the provincial BMA 6 boundary is the combination of the Recovery and Support Zones, grizzly bears occur outside the eastern boundary, and we included bears detected outside this eastern boundary in our analysis. Bears in BMA 6 are a small part of the much larger international Rocky Mountains subpopulation that includes the Northern Continental Divide Ecosystem (NCDE) in the U.S. [44, 45]. Human population density is approximately 0.9/km^2^.

There is an abrupt transition between public forested mountainous land to the west, and private agricultural land to the east. Approximately 40.9% of the landscape is forested (deciduous, coniferous, and mixed), while 22.0% of the landscape is agricultural land that is used for both livestock and crop production. In addition to grizzly bears, other large carnivores include cougars (*Puma concolor*), black bears (*U*. *americanus*), and wolves (*Canis lupus*). Available native prey species include mule deer (*Odocoileus hemionus*), white-tailed deer (*O*. *virginianus*), elk (*Cervus elaphus*), moose (*Alces alces*), and bighorn sheep (*Ovis canadensis*). With the exception of grizzly bears, all native carnivores and prey are considered secure within the province. Domestic prey including cattle (*Bos taurus*), and a small number of sheep (*Ovis aries*) and goats (*Capra hircus*) also are present.

## Methods

We used DNA extracted from hair samples to identify individual grizzly bears. We collected hair samples from natural rub objects established within the study area during 2011–2014. Rub objects included trees, power poles, fence posts, as well as stretches of barbed-wire fence the bears traveled along or through. The first two years of the project, 2011 and 2012, were primarily set-up years and included fewer visits to rub objects (2011: 2 visits Recovery Zone only, 2012: 8 visit Recovery Zone, 2 visits Support Zone). Then, during 2013 and 2014 rub objects in both the Recovery and Support Zones were visited 8 times (7 sampling occasions). Additionally, we opportunistically collected hair samples from agricultural lands within the Support Zone. Specifically, we worked with over 70 landowners as well as provincial Fish and Wildlife Officers to collect hair samples using standard protocols at grizzly bear incident sites as well as opportunistic observations on private lands. A full description of field sampling methods can be found in Morehouse and Boyce [29].

Hair samples were used to identify species, individual identity, and sex via analysis of nuclear DNA extracted from hair follicles following the protocols outlined by Paetkau [46, 47]. We used the G10J marker for species assignment to black bear versus grizzly bear [48]. Multi-locus genotyping followed Paetkau’s [47] 3-phase process of first pass, error check, and clean-up using the established 8-locus marker system (7 microsatellites plus the amelogenin marker for sex) for grizzly bears in the southern Rocky Mountain region [46, 49]. Because an insufficient number of loci is one of the primary causes of incorrect assignment in a parentage analysis [50, 51, 52, 53], we extended the genotypes of the known individuals to 24 microsatellite loci in 2014 after we had finished adding to our genetic dataset. We genotyped 213 unique grizzly bears (118 male, 95 female) from southwestern Alberta at 24 microsatellite loci, plus the amelogenin marker for sex.

In addition to insufficient loci, the other primary cause of incorrect parent assignment is incomplete sampling of candidate parents [50, 52, 54]. Because grizzly bears in southwestern Alberta are a small part of a larger international population that includes Montana and British Columbia [29, 44, 55], we included in our parentage analysis data from neighboring jurisdictions to increase the likelihood of identifying complete triads (mother, father, offspring). Over 50% of grizzly bears detected by our sampling methods had been previously genotyped by projects in Montana and British Columbia [29], and 16 bears had been previously genotyped by the 2007 Alberta inventory project [56]. Montana grizzly bear genetic data were obtained from previous non-invasive genetic sampling projects throughout the NCDE [57, 58, 59]. British Columbia grizzly bear genetic data were obtained from the B.C. Ministry of Forests, Lands, and Natural Resource Operations [60]. Additionally, we also obtained data on bears genotyped under a previous Alberta inventory [56]. Individuals were genotyped at 6 to 24 microsatellite loci (mean loci = 15.91, mode = 24). We used 2043 individual grizzly bears (977 males, 1072 females) from the Rocky Mountains subpopulation [44] in our parentage analysis. In 6 cases, sex was unknown and we analyzed those bears as both potential fathers and potential mothers.

We used program COLONY to assign parentage [61]. COLONY uses full pedigree likelihood methods to simultaneously infer sibship and parentage among individuals [61]. The likelihood is considered over the entire pedigree rather than for pairs of individuals. Simultaneously accounting for parent-offspring pairs as well as full- and half-sibs, increases accuracy of assignments [53, 62], and in a recent review, the full pedigree likelihood method implemented in COLONY outperformed other parentage methods [52]. We set the estimated proportion of parents in the dataset at 0.4 for each sex, and specified genotyping error at 0.001 based on error rates published by Wildlife Genetics International (Nelson, BC) [58]; COLONY is robust to these parameters provided sufficient information is contained in the data [62, 63]. Other specified parameters included: polygamous males and females, long run length (~1.9 billion iterations), full-likelihood analysis, and medium-likelihood precision. Ages were known for some bears (i.e. bears that were physically handled and age determined by cementum annuli), and we used this information to rule out potential parents if they were not at least 2 years older than other bears at that bear’s birth (n = 242). For example, if a bear was born in 2000 it was excluded as a parent for a bear born in 2002 or earlier, but was considered a potential parent for a bear born in 2003 or thereafter.

While we used the larger genetic data set for our parentage analysis to ensure our parentage assignments were as robust as possible, our analysis of problem bears used only the Alberta offspring data because our targeted sampling of grizzly bear incident and human-bear conflict locations was limited to southwestern Alberta. We define an incident as an occurrence where the grizzly bear caused property damage, obtained anthropogenic food, or killed or attempted to kill livestock or pets [32, 64]. Incident occurrences were grouped as property damage, livestock, attractant, and other [32]. A few additional samples associated with an incident came from areas outside the officially designated BMA 6 boundaries. We also included 4 hair samples associated with a human-bear conflict in which the bear made physical contact with a person, was killed by a person in self-defence, or, in one case, was deemed an aggressive bear and subsequently translocated by provincial Fish and Wildlife Officers to ensure public safety. We considered a bear a problem bear if it was associated with either an incident or human-bear conflict via detection by either non-invasively collected hair samples at incident sites, or from hair samples obtained by physical capture (done by provincial Fish and Wildlife Officers).

If the parent of an Alberta offspring was not contained in our Alberta data (i.e. was a bear detected only in Montana or British Columbia), we obtained conflict history from the respective state/province when possible. We classified all parent-offspring relationships as within-group pairs (PP-PO, PP-NPO, NPP-PO, NPP-NPO; PP = problem parent, PO = problem offspring, NPP = non-problem parent, NPO = non-problem offspring). COLONY assigns an “inferred” parent if the most likely genotype is not included in the input of candidate parents. We excluded these inferred mothers and fathers from our analysis because they were not actually detected by our sampling methods and thus their conflict status was unknown. Although we cannot determine age from hair, we assumed that if a female and her offspring were detected together at the same location on the same date, that the offspring were cubs and traveling with their mother. Consequently, any decisions regarding resource use were being made by their mother and not the offspring themselves. We excluded these observed mother-offspring detections from our analysis. Although it is possible that we included cases where an offspring was traveling with its mother and should have been excluded from our analysis, there is no reason to believe that our detections of mothers versus cubs should be biased one way or another. Any variations in detection of bears at incident or human-conflict sites should be random, and thus would not bias our results.

We used Barnard’s test [65, 66, 67] to compare the frequency of problem and non-problem offspring from problem and non-problem parents. First, to rule out the possibility of conflict behaviour being associated with a sex-linked gene, we used 4 sex-specific Barnard’s tests to evaluate mother-daughter, mother-son, father-daughter, and father-son relationships to compare the frequency of problem and non-problem sons and daughters from mothers and fathers. We would expect significant results in all 4 tests only if conflict behaviour is genetically sex-linked. Second, we considered each parent’s sex separately (i.e. one Barnard’s test for mothers, one Barnard’s test for fathers) to evaluate evidence for social learning. If social learning alone is present, we would expect a significant relationship for mother-offspring behaviours but not for father-offspring behaviours.

To evaluate the number of bears with potential exposure to conflict situations (e.g. a bear detected only in a remote area of public land would not be involved in an agricultural conflict), we evaluated the land tenure associated with each detection for each bear. In other words, we determined how many grizzly bears were detected exclusively on private land, exclusively on public land, and on both public and private lands. We determined these detection metrics both for all bears detected and the number of individuals associated with an incident or human-bear conflict site to help interpret our results.

All field methods were completed in accordance with the Canadian Council on Animal Care guidelines and approved by the University of Alberta BioSciences Animal Care and Use Committee (Protocol # AUP00000008). Field permits were granted by Alberta Environment and Parks, and Parks Canada.

## Results

From 2011 through 2014, we opportunistically collected 86 hair samples in Alberta that were associated with a grizzly bear incident (n = 82) or human conflict (n = 4) location; these 86 incident/human-bear conflict hair samples were assigned to 55 unique individuals (24 females, 31 males). Of the 213 identified grizzly bears from our broader sampling effort, 79 were detected exclusively on private land, 76 were detected exclusively on public land, and 58 were detected on both public and private lands. Of the 55 problem bears, 40 were detected exclusively on private land, and 15 on both public and private lands.

We evaluated a total of 76 mother-offspring and 119 father-offspring relationships. Our parentage analysis identified 28 unique mothers and 33 unique fathers within the Alberta data. We identified 61 mother-offspring and 88 father-offspring pairs for Alberta detected bears, but excluded 13 of the 61 mother-offspring relationships because they were situations in which the offspring were only detected with their mother. We included in our total 28 mother-offspring and 31 father-offspring relationships after obtaining parent conflict history from Montana. Montana conflict history changed the conflict status of 5 Alberta detected bears, bringing our total number of Alberta problem bears to 60 (out of 213). All offspring were bears detected in Alberta. Within the 76 mother-offspring relationships we evaluated, 30.3% (n = 23) of offspring were identified as problem bears independent of their mother’s conflict status. For the father-offspring relationships we evaluated, 28.6% (n = 34) of offspring were identified as problem bears independent of their father’s conflict status.

**Fig 2:**
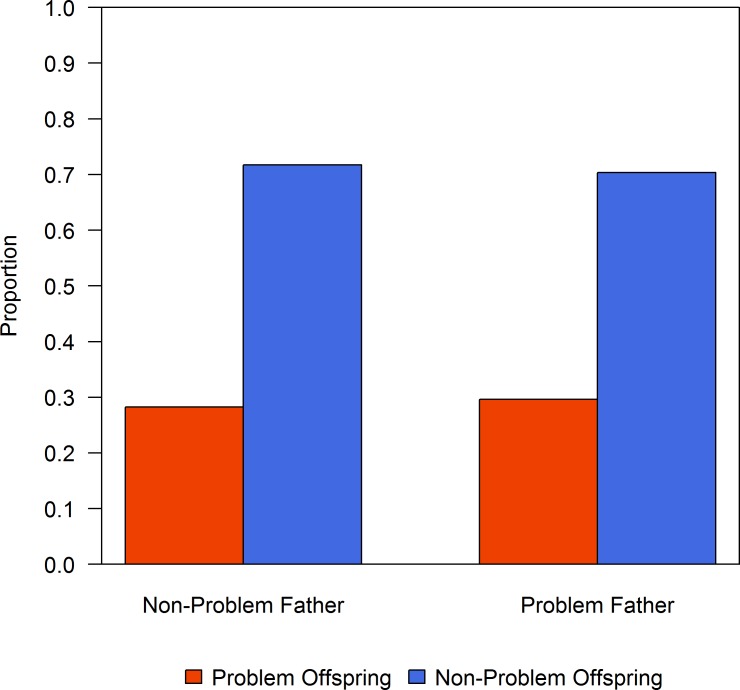
Father-offspring behaviours. Frequency of problem and non-problem offspring grouped by behaviour type of their father.

**Fig 3:**
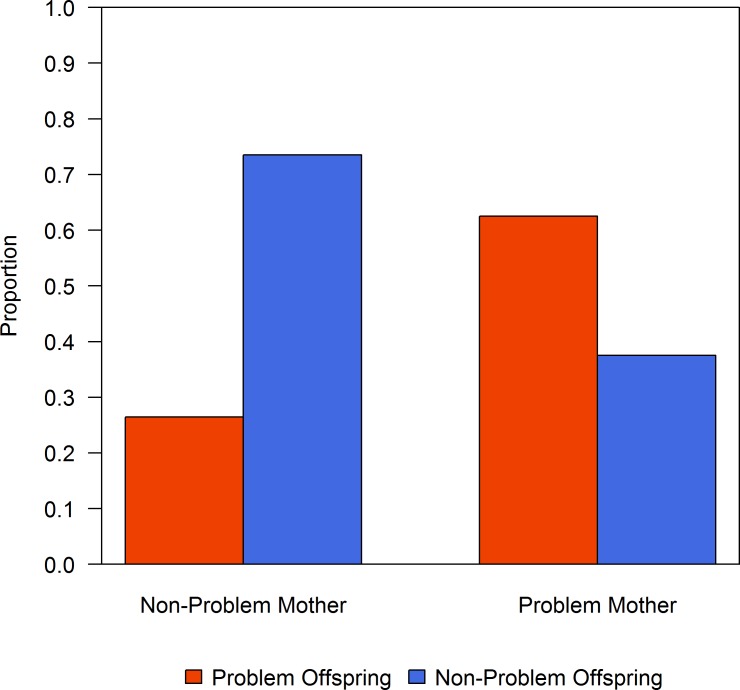
Mother-offspring behaviours. Frequency of problem and non-problem offspring grouped by behaviour type of their mother.

There was no evidence that conflict behaviour was associated with a sex-linked gene (Barnard’s test: mother-daughter, *p* = 0.17; mother-son, *p* = 0.12; father-daughter, *p* = 1.0; father-son, *p* = 0.83). There was no evidence that offspring were more likely to be involved in conflict behaviours when their fathers were problem bears (Barnard’s test, *p* = 0.92, 29.6% of offspring from problem fathers were problem bears, Fig 2). In contrast, offspring were more likely to be involved in incidents or human-bear conflict when their mothers were problem bears (Barnard’s test, *p* = 0.05, 62.5% of offspring from problem mothers were problem bears, Fig 3). There were 5 cases in which problem mothers had problem offspring; we were able to confirm that these 5 offspring were independent bears traveling separately from their mothers because these individuals were trapped by Fish and Wildlife officers who estimated their age and confirmed independence.

## Discussion

Our results provide evidence of a behavioural mechanism (i.e. social learning) that might be amplifying the propensity for grizzly bear-agricultural conflicts in southwestern Alberta. More offspring exhibited conflict behaviours when their mothers were problem bears, but no such effect was observed for paternal-conflict behaviour. Thus, our results support the social learning hypothesis, but not the genetic inheritance hypothesis as it relates to the acquisition of conflict behaviour. If human-bear conflict was an inherited behaviour, we would have expected to see a significant relationship between paternal conflict behaviour and offspring behaviour. The accuracy of our parentage assignments was increased by the large number of markers and the inclusion of a high proportion of candidate parents; thus, while it is possible that there were a small degree of errors in assignments, we believe our sampling and analysis methods have minimized potential errors. Despite the relatively low number of offspring from problem mothers (*n* = 8), our results provide more direct evidence for social learning in grizzly bears than previous studies (e.g. [22]) because we used a parentage analysis rather than relatedness to examine family relationships. Relatedness analyses cannot distinguish between full-sibling and parent-offspring relationships (e.g. relatedness coefficients for full-siblings and parent-offspring are both 0.5) [68]. Conclusions regarding social learning from relatedness analyses could therefore be influenced by other variables including philopatry in addition to social learning alone. The only concrete social learning interaction that can be evaluated with genetic data is the mother-offspring relationship assigned through a parentage analysis.

Social learning has the potential to perpetuate grizzly bear conflicts–highlighting the importance of preventing initial conflicts, but also removing problem individuals once conflicts start. Prompt removal (i.e. culling) of grizzly bears engaged in conflict behaviour might be an effective solution for reducing conflicts [69, 70], but removing females is unlikely to be a provincially approved mitigation measure in Alberta because grizzly bears have threatened status in the province. Indeed, one of the measures of success within the provincial Recovery Plan is to have no female grizzly bears killed as problem animals through agency control [35]. However, problem grizzly bears in Alberta often are relocated (moved within the BMA) or translocated (moved outside the BMA), and relocation/translocation is completed according to the provincial grizzly bear response guidelines [36]. While male grizzly bears can be relocated long distances outside of the bear management area, it is mandatory on a first offence that a female grizzly bear with cubs stays within the same BMA [36].

Further, long-distance relocation of problem individuals often is unsuccessful (e.g. [42, 69, 71, 72]), and translocated bears typically have higher mortality rates and lower survival than non-translocated bears [41, 42]. Within Alberta the overall success rate of translocations and relocations of problem grizzly bears is only 30.5%, with translocated individuals re-offending, homing, or suffering increased mortality [73]. Thus, Alberta’s bear management policy might be increasing mortality risk for males disproportionately to females. At the same time, because of social learning in conflict behaviour, keeping females within the same bear management area might be providing more learning opportunities for cubs of problem females, and consequently increasing the number of problem bears.

However, not every bear that uses private lands will become a problem bear. Indeed, of the 213 grizzly bears detected, 137 were detected on private land at some point, but only 55 grizzly bears were identified as problem bears (an additional 5 bears were involved in incidents in Montana). Emphasizing proactive (e.g. attractant management) rather than reactive (e.g. relocation) mitigation measures might be a more effective long-term solution [71], and unlike lethal removal, such proactive efforts fit within the objectives of Alberta’s grizzly bear Recovery Plan, and also are likely to be more cost effective than translocation. Not every problem bear will remain a problem bear; for example, an individual bear might access anthropogenic food resources only when natural food resources are scarce (e.g. [26]). While accessing anthropogenic resources such as dead stock in a bone pile could be considered natural grizzly bear foraging behaviour, such behaviour is not desirable on private lands. Securing anthropogenic food sources through attractant management can be a powerful tool for preventing conflicts (e.g. [74, 75]), and local community groups in southwestern Alberta have demonstrated effective mitigation measures (e.g. electric fencing, grain bin retrofits, dead stock removal, etc.) [32].

Additionally, aversive conditioning can be an effective strategy for preventing bears from developing undesirable behaviours [70, 76, 77]. Aversive conditioning uses a negative stimulus to cause pain, discomfort, or irritation in an animal involved in an unwanted behaviour [76, 77, 78]. Aversive conditioning, however, will not be an effective management tool if unsecured attractants remain in the area [76, 77, 79]. Thus, attractant management likely should be prioritized over aversive conditioning.

Such mitigation measures are important because the area has an increasing grizzly bear population that overlaps substantially with agricultural land uses [29]. The most recent abundance estimate for our study area is 67 resident bears, and the population is estimated to be growing at 4% per year [29]. However, far more bears use the study area than are considered resident bears; the estimate of grizzly bears using the study area during the course of a year is 172 and this number represents the number of bears that have the potential to be involved in conflict [29]. Increased collaboration across jurisdictional boundaries will likely improve both research and management of this international and interprovincial grizzly bear population.

While Galton advocated an “inheritance of criminal tendencies” in humans [1], we found no evidence for a genetic basis for “criminal” behaviour by bears. We might, however, be able to prevent learning of conflict behaviour by minimizing opportunities for females to become problem bears and quickly addressing and/or removing the source of the conflict once discovered. Because grizzly bears are provincially threatened, lethal removal of problem female grizzly bears is a last resort [36]. Thus, preventing conflict behaviours through proactive initiatives such as electric fencing, attractant management, grain bin modifications, and potentially aversive conditioning offer more promising solutions that both reduce the economic impact of grizzly bears to producers but could also help stop the acquisition of conflict behaviours. While it is possible we might have misclassified the behaviour of some bears (e.g., Fish and Wildlife Officers could have captured a bear at a conflict site but accidentally caught the wrong bear), there was a strong pattern of non-problem mothers (*n* = 68) producing non-problem offspring. Additional studies would further test whether this social learning hypothesis is supported in other circumstances and populations of bears. In the meantime, preventing female grizzly bears from becoming problem individuals will likely help prevent the perpetuation of conflicts through social learning.

## Supporting Information

S1 Data File Contingency tables of grizzly bear behaviour. (XLSX)
